# Geometric Algebra-Based ESPRIT Algorithm for DOA Estimation

**DOI:** 10.3390/s21175933

**Published:** 2021-09-03

**Authors:** Rui Wang, Yue Wang, Yanping Li, Wenming Cao, Yi Yan

**Affiliations:** 1 School of Communication and Information Engineering, Shanghai University, Shanghai 200444, China; rwang@shu.edu.cn (R.W.); wangyue8@shu.edu.cn (Y.W.); yanpingli@shu.edu.cn (Y.L.); 2 College of Information Engineering, Shenzhen University, Shenzhen 518060, China; 3 National Space Science Center, Chinese Academy of Sciences, Beijing 100190, China; yanyi@nssc.ac.cn

**Keywords:** direction-of-arrival estimation, geometric algebra, ESPRIT algorithm, electromagnetic vector-sensor array

## Abstract

Direction-of-arrival (DOA) estimation plays an important role in array signal processing, and the Estimating Signal Parameter via Rotational Invariance Techniques (ESPRIT) algorithm is one of the typical super resolution algorithms for direction finding in an electromagnetic vector-sensor (EMVS) array; however, existing ESPRIT algorithms treat the output of the EMVS array either as a “long vector”, which will inevitably lead to loss of the orthogonality of the signal components, or a quaternion matrix, which may result in some missing information. In this paper, we propose a novel ESPRIT algorithm based on Geometric Algebra (GA-ESPRIT) to estimate 2D-DOA with double parallel uniform linear arrays. The algorithm combines GA with the principle of ESPRIT, which models the multi-dimensional signals in a holistic way, and then the direction angles can be calculated by different GA matrix operations to keep the correlations among multiple components of the EMVS. Experimental results demonstrate that the proposed GA-ESPRIT algorithm is robust to model errors and achieves less time complexity and smaller memory requirements.

## 1. Introduction

Direction-of-arrival (DOA) estimation of electromagnetic (EM) signals has attracted wide attention in many communication fields, such as radar [1,2], mobile networks [3] and sonar [4]. It is clear that DOA estimation is the basic and essential part in an array signal processing system. For example, a corresponding transmitting or receiving beamformer can be designed to extract signals in the direction of interest and suppress uninteresting interference signals. The electromagnetic vector sensor (EMVS) can catch polarization-related information compared to a conventional scalar sensor, which can further improve the target resolution, anti-interference ability and detection stability for DOA estimation [5,6,7]; therefore, the research for EMVS array direction finding has become a hotspot.

With the appearance of the Long-Vector MODEL (LV-MODEL) [5] (built for EMVS), multiple researchers have proposed various DOA estimators. The existing estimators can be summarized into three categories: (1) research on DOA estimators transplanting from scalar sensor; (2) research based on special array arrangement; (3) research based on advanced mathematical tools.

In terms of transplantation, the classic subspace-based super-resolution algorithm [8] (Multiple Signal Classification—MUSIC) was transplanted to the EMVS [9,10,11] array, but the algorithms often suffer high computational complexity because of the four-dimensional parameter search for two direction angles and two additional polarization angles; therefore, Weiss [12] used the polynomial root to reduce the computational complexity to a certain extent. In addition, another subspace-based super-resolution algorithm [13,14] (Estimation of Signal Parameters via Rotational Invariance Techniques—ESPRIT) was also transplanted into the EMVS array, and realized closed-form estimation of DOA. In [15,16], authors showed that the statistical performance of the maximum likelihood and subspace-fitting algorithms based on the EMVS array are better than both MUSIC and ESPRIT, but the high calculation limits its application in actual engineering.

There are few studies based on the special array arrangement because most EMVS arrays are co-centered, leading to the mutual coupling interference and spatial information loss. In [17], a double-parallel-line EMVS array whose six components are all spatially separated achieved mutual coupling reduction to refine the DOA-finding accuracy by orders of magnitude. A triangular array [18] combined with a vector cross product and interferometric angle measurement, aimed to overcome the drawback that [17] cannot achieve two-dimensional aperture expansion. In addition, a spatial expansion method of a triangle structure [19] was proposed to provide higher-precision DOA estimation.

The traditional model for EMVS is just a linear combination of each component, which somehow locally destroy the orthogonality of the signal components [20]. Meanwhile, the heavy computational efforts and memory requirements during data processing for the DOA estimation cannot be ignored [21]. Recently, the hypercomplex has been widely studied and applied in multi-dimensional parameter estimation. Miron et al. [22] first proposed a new Quaternion Model (Q-MODEL) for the two-component EMVS array. Then, many models and algorithms based on quaternion have been proposed [23,24,25]; however, the Q-MODEL had to discard some of the original information because the quaternion only has three imaginary parts. Further, the research has extended to bi-quaternion [26] and quad-quaternion [27,28]. These quaternion-based algorithms showed higher estimation accuracy and less complexity; however, Jiang et al. [21] found that the physical interpretations of the presented quaternion-like models have not been discussed. In order to solve the problem, they derived G-MODEL [21] by Geometric Algebra (GA) formulations of Maxwell equations. The computing technology of G-MODEL not only minimizes the memory requirements and computational complexity, but also removes the correlation of noise on different antennas.

It is easy to find that the current studies utilizing hypercomplex algebra are mainly focused on the MUSIC algorithm [22,26,27,28]. In fact, MUSIC greatly suffers from a heavy computational burden for its spectrum search, while the computation of ESPRIT algorithm is cheaper, and it can automatically decouple [29]; therefore, the research in this paper extends the ESPRIT algorithm using a new mathematical tool—GA. Through the new calculation rules, the physical nature of EMVS is matched with the signal processing technology, which avoids correlation loss between different components in the previous algorithms. The major contributions of this paper are as follows.

1. We incorporate the multi-dimensional consistency of GA into ESPRIT, and propose a Geometric Algebra-based ESPRIT algorithm (GA-ESPRIT) for 2D-DOA estimation.
2. We use the new calculation rules of the high-dimensional algebra system to preserve the correlation among multiple components of EMVS.
3. Experimental results demonstrate that the proposed GA-ESPRIT algorithm can achieve more accurate, stable and lighter DOA estimation.

The rest of this paper is organized as follows. Section 2 introduces the basics of GA and the EMVS model for narrow-band signals based on GA. Section 3 describes the proposed GA-ESPRIT in detail. Experimental results and analysis are provided in Section 4, followed by concluding remarks in Section 5.

## 2. Preliminaries

### 2.1. Fundamental of Geometric Algebra

The concept of GA [30] was proposed by David Hestenes in the 1960s, who combined Clifford Algebra with a physical geometric structure. After decades of research, GA has shown its absolute superiority in electromagnetism [31], cosmology [32], multi-channel image [33,34,35] and other physical sciences.

#### 2.1.1. Geometric Product

The crucial product operation in GA theory is the geometric product [30]. For vectors a and b, the geometric product is denoted by

(1) ab=a·b+a∧b,

where {·} and {∧} denote the inner product and the outer product, respectively.

#### 2.1.2. Multi-Vector

Let $G_{n}=C\ell_{n,0}$, which is the real GA of the quadratic pair (V,Q) where $V=R^{n}$ and *Q* is the quadratic form of signature (n,0). There is an orthogonal basis $\left\{e_{1},e_{2},\ldots ,e_{n}\right\}$ in $R^{n}$, which generates $2^{n}$ basis elements of $G_{n}$ via the geometric product as shown in (2):

(2) $$\underset{k=0}{\underset{︸}{\left\{1\right\}}},\underset{k=1}{\underset{︸}{\left\{e_{i}\right\}}},\underset{k=2}{\underset{︸}{\left\{e_{ij},i<j\right\}}},\ldots ,\underset{k=n}{\underset{︸}{\left\{e_{1}e_{2}\cdots e_{n}\right\}}}$$

for i,j=1,2,…,n.

The multi-vector *A* of $G_{n}$ is defined as

(3) $$\begin{array}{cc} A & =E_{0}\left(A\right)+\sum_{1\leq i\leq n}E_{i}\left(A\right)e_{i}+\sum_{1\leq i<j\leq n}E_{ij}\left(A\right)e_{ij}+\ldots +E_{1\ldots n}\left(A\right)e_{1\ldots n}\\ \; & ={\langle A\rangle }_{0}+{\langle A\rangle }_{1}+{\langle A\rangle }_{2}+\ldots +{\langle A\rangle }_{n}, \end{array}$$

where $E_{i}\left(A\right),E_{ij}\left(A\right),\ldots ,E_{1\ldots n}\left(A\right)\in R$, and ${\langle A\rangle }_{k}$ denotes the component of *A* of grade *k*.

The reverse of multi-vector *A* is defined as

(4) $$\tilde{A}=\sum_{k=0}^{n}{\left(-1\right)}^{k(k-1)/2}{\langle A\rangle }_{k}.$$

### 2.2. The Geometric Algebra of Euclidean 3-Space

According to the structural characteristics of EMVS, $G_{3}$ is chosen to model and process the received signals [21]. The multiplication rule can be found in Table 1.

Referring to (2) and (3), a $G_{3}$ matrix with *m*-row and *n*-column, noted $G_{3}^{m\times n}$, is constructed as follows [20]

(5) $$\begin{array}{cc} A & =A_{0}+A_{1}e_{1}+A_{2}e_{2}+A_{3}e_{3}+A_{4}e_{12}\\ & +A_{5}e_{23}+A_{6}e_{13}+A_{7}e_{123}, \end{array}$$

where $A_{k}$ for k=1,2,3,…,7 are all m×n real number matrices. The transpose with reversion of A is denoted by $A^{H}$

(6) $$\begin{array}{cc} A^{H} & =A_{0}^{T}+A_{1}^{T}e_{1}+A_{2}^{T}e_{2}+A_{3}^{T}e_{3}-A_{4}^{T}e_{12}\\ & -A_{5}^{T}e_{23}-A_{6}^{T}e_{13}-A_{7}^{T}e_{123}, \end{array}$$

where $A_{i}^{T}$ for k=1,2,3,…,7 denotes the transpose.

### 2.3. G-MODEL

A compact polarized GA model for the vector-sensor array was proposed in [21], named G-MODEL, which models the six-component outputs of a vector sensor holistically using a multi-vector in $G_{3}$. Suppose there are *K* narrow-band, far-field and uncorrelated sources with wavelength λ impinging on an array, which includes *Q* vector sensors. Define $\theta_{k}\in \left[0,2\pi \right)$, $\phi_{k}\in \left[0,\pi \right)$, $\gamma_{k}\in \left[0,\pi /2\right)$ and $\eta_{k}\in \left[-\pi ,\pi \right)$ are the azimuth angle, elevation angle, polarization amplitude angle and phase difference angle of the kth source, respectively.

**Figure 1:**
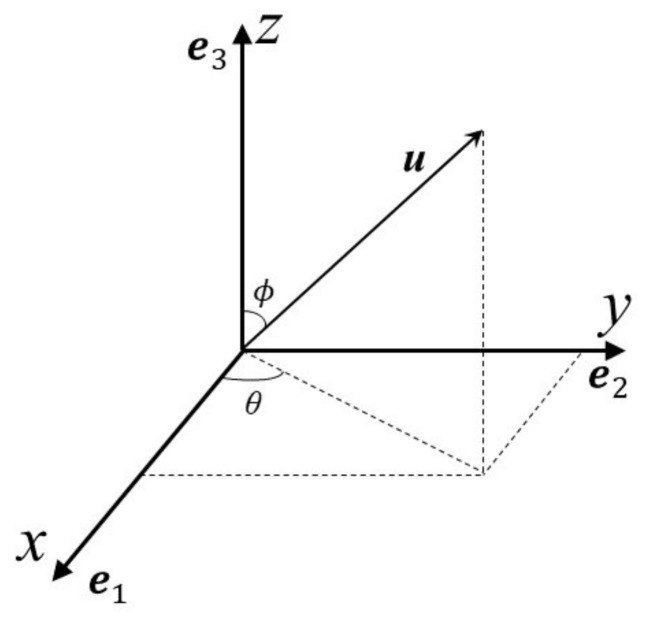
Direction vector of incident source.

Define $u_{k}=\cos\theta_{k}\sin\phi_{k}e_{1}+\sin\theta_{k}\sin\phi_{k}e_{2}+\cos\phi_{k}e_{3}$ as the unit vector (see Figure 1) of the kth source when it impinges on the sensor at the origin. $v_{k1}=-\sin\theta_{k}e_{1}+\cos\theta_{k}e_{2}$ and $v_{k2}=\cos\theta_{k}\cos\phi_{k}e_{1}+\sin\theta_{k}\cos\phi_{k}e_{2}-\sin\phi_{k}e_{3}$ are unit multi-vectors. The position vector of the qth sensor is $r_{q}=r_{q1}e_{1}+r_{q2}e_{2}+r_{q3}e_{3}$. The output of the qth vector sensor in the array is denoted by [21]

(7) $$Y_{EH}^{\left(q\right)}\left(t\right)=\sum_{k=1}^{K}X_{q}\left(\theta_{k},\phi_{k}\right)V_{k}P_{k}S_{k}\left(t\right)+N_{EH}^{\left(q\right)}\left(t\right),$$

where $X_{q}\left(\theta_{k},\phi_{k}\right)=e^{e_{123}\frac{2\pi }{\lambda }\left(\cos\theta_{k}\sin\phi_{k}r_{q1}+\sin\theta_{k}\sin\phi_{k}r_{q2}+\cos\phi_{k}r_{q3}\right)}$ is the spatial phase factor of the kth source incident on the qth vector sensor.

$$\begin{array}{cc} & V_{k}=\left(1+u_{k}\right)\left[v_{k1},-v_{k2}\right],\\ & P_{k}=\left[\begin{array}{c} \cos\gamma_{k}\\ \sin\gamma_{k}e^{e_{123}\eta_{k}} \end{array}\right],\\ & S_{k}\left(t\right)=\left|S_{k}\left(t\right)\right|\exp\left[e_{123}\left(2\pi f_{k}t\right)\right]. \end{array}$$

In next section, the GA-ESPRIT algorithm is deduced based on the G-MODEL.

## 3. Proposed Algorithm

The basic premise of the ESPRIT algorithm is that there are identical subarrays, the spacing between subarrays is known and the structure of subarrays is identical, which satisfies the rotational invariance in space [13]. Uniform linear arrays (ULAs) appear when it comes to one-dimensional DOA estimation using conventional ESPRIT [1,13]. Compared with ULAs, double parallel uniform linear arrays (DPULAs) can identify two-dimensional DOA because of the special construction, which consists of two parallel ULAs [36,37,38]; therefore, the algorithm discussed in this paper is based on DPULAs.

### 3.1. Complex Representation Matrix and Related Calculations

In view of the paucity of research on calculations with multi-vector, the Complex Representation Matrix (CRM) [20] is introduced because of the mature matrix theories. Consider a matrix $A\in G_{3}^{m\times n}$, the CRM is defined by Ψ(A)

(8) $$\Psi \left(A\right)=\left(\begin{array}{cc} A_{0}+A_{3}+\left(A_{7}+A_{4}\right)e_{123} & -A_{1}+A_{6}+\left(A_{2}-A_{5}\right)e_{123}\\ -A_{1}-A_{6}-\left(A_{2}+A_{5}\right)e_{123} & A_{0}-A_{3}+\left(A_{7}-A_{4}\right)e_{123} \end{array}\right).$$

Let $\nu =\left(-e_{1}+e_{13}\right)/2\in G_{3}$, and its reversion is $\tilde{\nu }=\left(-e_{1}-e_{13}\right)/2\in G_{3}$. Then,

(9) $$\nu^{2}={\tilde{\nu }}^{2}=0\;\;\mathrm{and}\;\;\nu \tilde{\nu }+\tilde{\nu }\nu =1,$$

which imply $\nu \tilde{\nu }\nu =\nu$, $\tilde{\nu }\nu \tilde{\nu }=\tilde{\nu }$, ${\left(\nu \tilde{\nu }\right)}^{2}=\nu \tilde{\nu }$, ${\left(\tilde{\nu }\nu \right)}^{2}=\tilde{\nu }\nu$.

It immediately follows that, for every $A\in G_{3}$, we have

(10) $$A=E_{2m}\Psi \left(A\right)E_{2n}^{H},$$

(11) $$\Psi \left(A\right)=Q_{2m}\left[\begin{array}{cc} A & 0\\ 0 & A \end{array}\right]Q_{2n},$$

where in (10) and (11) we have

(12) $$E_{2k}=\left[\nu \tilde{\nu }I_{k}\;\tilde{\nu }I_{k}\right]\in G_{3}^{k\times 2k},$$

(13) $$Q_{2k}=\left[\begin{array}{cc} \nu \tilde{\nu }I_{k} & \tilde{\nu }I_{k}\\ \nu I_{k} & \tilde{\nu }\nu I_{k} \end{array}\right]\in G_{3}^{2k\times 2k}.$$

$I_{k}$ denotes the k×k identity matrix. It is not difficult to prove that

(14a) $$Q_{2k}=Q_{2k}^{H}={Q_{2k}}^{-1},$$

(14b) $$\Psi \left(A^{H}\right)={\left(\Psi (A)\right)}^{H},$$

(14c) $$\Psi \left(A^{+}\right)={\left(\Psi \left(A\right)\right)}^{+},$$

where {+} denotes the pseudo-inverse. Referring to (10) and (14c), the pseudo-inverse of any $A\in G_{3}$ is

(15) $$A^{+}=E_{2n}{\left(\Psi \left(A\right)\right)}^{+}E_{2m}^{H}.$$

Since $e_{123}^{2}=-1$ and $e_{123}$ commutes with all elements in $G_{3}$, one can identify it with the complex imaginary unit *j* [20], and so we can view Ψ(A) given in (8) as a complex matrix.

### 3.2. Model for DPULAs

**Figure 2:**
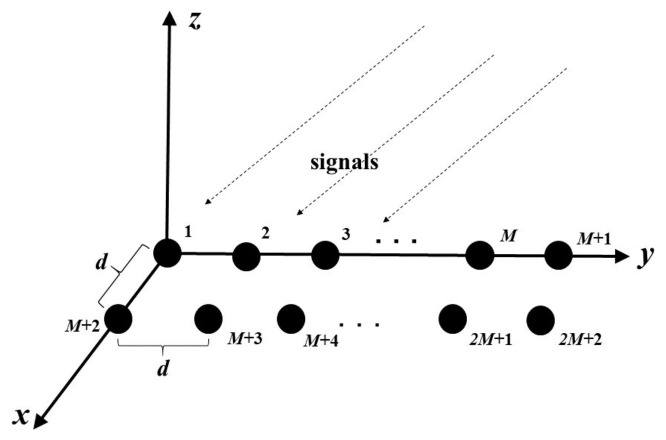
Double parallel uniform linear array.

**Figure 3:**
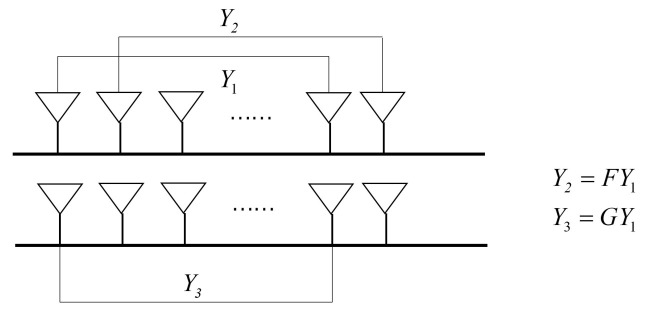
Schematic diagram of GA-ESPRIT.

Consider a DPULA with 2M+2 sensors, as shown in Figure 2, in which *d* and *M* refer to the spacing between two adjacent sensors and the number of sensors in per subarray, respectively. The array is divided into three subarrays. The 1st to *M*th sensors on the *x*-axis compose the first subarray, the 2nd to (M+1)th sensors form the second subarray and the (M+2)th to (2M+1)th that located on a straight line parallel to the *x*-axis make up the third subarray. The reason for the division can be found in Figure 3, that is, there are two unknown DOA parameters in the model, which need two rotational invariance relations.

Since the three subarrays have the same structure and the same number of sensor, each output of them has only one phase difference for the same signal. Signals received by subarray one, two and three are defined as $Y_{EH}^{1}$, $Y_{EH}^{2}$ and $Y_{EH}^{3}$, respectively. According to the above array model, the outputs of the three subarrays at time *t* are as follows

(16) $$\begin{array}{cc} Y_{EH}^{1}\left(t\right) & =AS\left(t\right)+N^{1}\left(t\right),\\ Y_{EH}^{2}\left(t\right) & =AFS\left(t\right)+N^{2}\left(t\right),\\ Y_{EH}^{3}\left(t\right) & =AGS\left(t\right)+N^{3}\left(t\right), \end{array}$$

where

(17) $$\begin{array}{cc} \; & Y_{EH}^{1}\left(t\right)={\left[Y_{EH}^{\left(1\right)}\left(t\right),\ldots ,Y_{EH}^{\left(M\right)}\left(t\right)\right]}^{T},\\ \; & Y_{EH}^{2}\left(t\right)={\left[Y_{EH}^{\left(2\right)}\left(t\right),\ldots ,Y_{EH}^{\left(M+1\right)}\left(t\right)\right]}^{T},\\ \; & Y_{EH}^{3}\left(t\right)={\left[Y_{EH}^{\left(M+2\right)}\left(t\right),\ldots ,Y_{EH}^{\left(2M+1\right)}\left(t\right)\right]}^{T}, \end{array}$$

and

(18) $$\begin{array}{cc} \; & A=\left[a\Gamma_{1},\ldots ,a\Gamma_{K}\right],\\ \; & a\left(\Gamma_{k}\right)={\left[1,x\left(\theta_{k},\phi_{k}\right),\ldots ,x^{M-1}\left(\theta_{k},\phi_{k}\right)\right]}^{T}V_{k}P_{k},\\ \; & x\left(\theta_{k},\phi_{k}\right)=e^{e_{123}\frac{2\pi }{\lambda }d\cos\theta_{k}\sin\phi_{k}},\\ \; & F=\mathrm{diag}\left(f_{1},\ldots ,f_{K}\right),\;G=\mathrm{diag}\left(g_{1},\ldots ,g_{K}\right). \end{array}$$

According to (18), we find that the DOA information is contained in matrix A, F and G. Because F and G are diagonal matrices that only contain direction information of incident signals, the focus is the two matrices, i.e.,

(19) $$\begin{array}{c} f_{k}=e^{e_{123}\frac{2\pi }{\lambda }d\cos\theta_{k}\sin\phi_{k}},\\ g_{k}=e^{e_{123}\frac{2\pi }{\lambda }d\sin\theta_{k}\sin\phi_{k}}. \end{array}$$

Clearly, it is easy to figure out the DOA in the light of (19) if we obtain the two ideal matrices F and G. From the rules of subarray division, we can see that the latter (M−1) sensors of subarray one and the former (M+1) sensors of subarray two are overlapped. Thus, in order to reduce the computational complexity, subarray one and subarray two can be merged to form a new matrix $Y_{EH}$, that is,

(20) $$Y_{EH}\left(t\right)={\left[y_{1}\left(t\right),y_{2}\left(t\right),\ldots ,y_{M+1}\left(t\right)\right]}^{T}.$$

After merging, the (2M+2)th redundant sensor is added to subarray three to form a new subarray $P_{EH}$, so that the third subarray has the same dimension as $Y_{EH}$

(21) $$P_{EH}\left(t\right)={\left[y_{M+2}\left(t\right),y_{M+3}\left(t\right),\ldots ,y_{2M+2}\left(t\right)\right]}^{T}.$$

Let $\bar{A}$ be the array flow pattern of $Y_{EH}$, then

(22) $$\begin{array}{c} \bar{A}=[\bar{a}(\Gamma_{1}),\bar{a}(\Gamma_{2}),\ldots ,\bar{a}(\Gamma_{K})],\\ \bar{a}(\Gamma_{k})={\left[1,x\left(\theta_{k},\phi_{k}\right),\ldots ,x^{M}\left(\theta_{k},\phi_{k}\right)\right]}^{T}V_{k}P_{k}. \end{array}$$

$Y_{EH}$ and $P_{EH}$ can be written as

(23) $$\begin{array}{cc} Y_{EH}\left(t\right) & =\bar{A}S\left(t\right)+N_{a}\left(t\right),\\ P_{EH}\left(t\right) & =\bar{A}GS\left(t\right)+N_{b}\left(t\right), \end{array}$$

where

$$\begin{array}{c} N_{a}\left(t\right)=\left[\begin{array}{c} N^{1}\left(t\right)\\ n_{M+1}\left(t\right) \end{array}\right],\;N_{b}\left(t\right)=\left[\begin{array}{c} N^{3}\left(t\right)\\ n_{2M+2}\left(t\right) \end{array}\right]. \end{array}$$

Then, B(t) is defined as

(24) $$B\left(t\right)=\left[\begin{array}{c} Y_{EH}\left(t\right)\\ P_{EH}\left(t\right) \end{array}\right]=CS\left(t\right)+N\left(t\right),$$

where

$$\begin{array}{c} C=\left[\begin{array}{c} \bar{A}\\ \bar{A}G \end{array}\right],\;N\left(t\right)=\left[\begin{array}{c} N_{a}\left(t\right)\\ N_{b}\left(t\right) \end{array}\right]. \end{array}$$

Finally, the output of the whole array is denoted by

(25) B(t)=CS(t)+N(t).

### 3.3. Algorithm Details

It is assumed that the sources received by the vector-sensor array are random signals which are independent and uncorrelated. In the same way, the measuring noise on six antennas of each sensor is white noise with the same power.

#### 3.3.1. Subspace Separation

Under the above assumption, theoretically, the covariance matrix of the array output is given by

(26) $$R=E\left\{BB^{H}\right\}=CR_{s}C^{H}+6\sigma^{2}I_{2M+2},$$

where E{·} stands for the mathematical expectation operator, $\sigma^{2}$ is the noise power on each vector antenna, $R_{S}=E\left\{S\left(t\right)S^{H}\left(t\right)\right\}$.

Since the geometric product is non-commutativity, the Eigenvalue Decomposition (ED) is different from the conventional real methods but similar to the quaternion case. In other words, there are two possible eigenvalues, namely the left and the right eigenvalue for $G_{3}$ matrix. In the proposed algorithm, the right eigenvalue is selected because the right ED of $G_{3}$ matrix can be converted to the right ED of its CRM [20].

The ED of R is denoted by

(27) $$R=U_{s}\Sigma_{s}U_{s}^{H}+U_{n}\Sigma_{n}U_{n}^{H}.$$

According to the principle of subspace separation, $U_{s}$ is the signal subspace corresponding to *K* larger eigenvalues, and $\Sigma_{s}$ is a diagonal matrix composed of *K* larger eigenvalues. In addition, $U_{n}$ is orthogonal to $U_{s}$ and it is the noise subspace corresponding to the remaining 4(M+1)−K small eigenvalues. Similarly, $\Sigma_{n}$ is a diagonal matrix composed of the remaining small eigenvalues.

In the actual processing, the received signal is usually sampled. So, for a certain number of snapshots *N*, (26) and (27) can be rewritten as

(28) $$\begin{array}{cc} \; & \hat{R}=\frac{1}{N}\sum_{i=1}^{N}B\left(t_{i}\right)B^{H}\left(t_{i}\right),\\ \; & \hat{R}={\hat{U}}_{s}{\hat{\Sigma }}_{s}{\hat{U}}_{s}^{H}+{\hat{U}}_{n}{\hat{\Sigma }}_{n}{\hat{U}}_{n}^{H}. \end{array}$$

Because the space formed by the eigenvectors corresponding to the larger eigenvalues is the same as the space formed by the steering multi-vectors of the incident signals, that is, $\mathrm{span}\left\{U_{s}\right\}=\mathrm{span}\left\{C\right\}$, there exists a unique non-singular matrix T, which satisfies

(29) $$U_{s}=CT.$$

The rotational invariance relations exist among three subarrays, but $U_{s}$ is the signal subspace of the whole array; therefore, after obtaining $U_{s}$, the signal subspace of three subarrays must be separated. By the arrangement of sensor array, we find that the signal subspace of three subarrays can be calculated by

(30) $$\begin{array}{cc} U_{s1} & =K_{1}U_{s}=CT,\\ U_{s2} & =K_{2}U_{s}=CFT,\\ U_{s3} & =K_{3}U_{s}=CGT, \end{array}$$

where $U_{s1}$, $U_{s2}$ and $U_{s3}$ are signal subspaces of subarray one, subarray two and subarray three, respectively.

(31) $$\begin{array}{cc} K_{1} & ={\left[\begin{array}{cc} I_{M} & 0_{M\times (M+2)} \end{array}\right]}_{M\times (2M+2)},\\ K_{2} & ={\left[\begin{array}{ccc} 0_{M\times 1} & I_{M} & 0_{M\times (M+1)} \end{array}\right]}_{M\times (2M+2)},\\ K_{3} & ={\left[\begin{array}{ccc} 0_{M\times (M+1)} & I_{M} & 0_{M\times 1} \end{array}\right]}_{M\times (2M+2)}. \end{array}$$

#### 3.3.2. Rotation Invariance

From (30), the pivotal matrices F and G can be found. So, let

(32) $$U_{s2}=U_{s1}\Psi_{x}$$

in the same way,

(33) $$U_{s3}=U_{s1}\Psi_{y}$$

It is discovered that the eigenvalues of $\Psi_{x}$ and $\Psi_{y}$ are diagonal elements of F and G, respectively.

Equations (32) and (33) are equations themselves, and are usually solved by the Least Squares (LS) method [29,36,38,39]; however, LS only takes the error on the left side of the equation into account, it ignores that the coefficient matrix also has an error; therefore, in order to reduce the error caused by solving the equation as much as possible, this paper considers a more accurate method—TLS [13]. Next, the solution of the equation is obtained by taking (32) as an example.

Combining the idea of TLS with the orthogonal property of subspace, we define a new matrix $U_{s12}=\left[U_{s1}\;U_{s2}\right]$. In fact, the main aim is to seek a unitary matrix $D\in G_{3}^{M\times 2K}$, which is orthogonal to $U_{s12}$. In other words, the space formed by D is orthogonal to the space formed by the column vectors of $U_{s1}$ or $U_{s2}$. So the D can be obtained from the ED of $U_{s12}^{H}U_{s12}$ [40]

(34) $$U_{s12}^{H}U_{s12}=E\Lambda E^{H},$$

where Λ is the diagonal matrix whose diagonal elements are composed by *K* multi-vectors that only have 0-grade-vector (can regard as non-zero real number) and 3K multi-vectors that equal to 0. E can be written as

(35) $$E=\left[\begin{array}{cc} E_{11} & E_{12}\\ E_{21} & E_{22} \end{array}\right].$$

Let $E_{N}=\left[\begin{array}{c} E_{12}\\ E_{22} \end{array}\right]$, which is composed by eigenvectors whose eigenvalues are 0 and form the noise subspace. Since $U_{s12}$ is signal subspace, we find that $D=E_{N}$, i.e.,

(36) $$U_{s12}D=\left[U_{s1}\;U_{s2}\right]\left[\begin{array}{c} E_{12}\\ E_{22} \end{array}\right]=0.$$

Then,

(37) $$\Psi_{x}=-E_{12}E_{22}^{+}.$$

The pseudo-inverse of $G_{3}$ matrix $E_{22}$ can be found in (15).

#### 3.3.3. Angle Estimation

The azimuth and elevation angle of *K* signals are included in F and G. In theory, the eigenvectors obtained by ED of these two matrices are both T; however, in the actual calculation process, the two eigenvalue decomposition operations are carried out independently, which can not ensure that the arrangement of eigenvectors in them is reflected well; therefore, the diagonal elements of F and G should be matched.

Suppose that T1 and T2 are eigenvector matrices derived from GA-ED of $\Psi_{x}$ and $\Psi_{y}$, respectively. Then

(38) $$O=|T2^{H}T1|$$

where {|·|} is the operator that gets magnitude of every multi-vector in a matrix. For the same signal, the eigenvectors in T1 and T2 corresponding to matched $f_{k}$ and $g_{k}$ are related; therefore, the order of diagonal elements in F and G can be adjusted by the coordinate of the largest element in each row (or column) of O to complete matching.

After observing (19), *f* and *g* are multi-vectors that only have scalar and 3-grade-vector, if we replace $e_{123}$ with the imaginary unit *j* of complex number, *f* and *g* can be regarded as complex numbers. Finally, we calculate $\theta_{k}$ and $\phi_{k}$ with $f_{k}$ and $g_{k}$, that is,

(39) $$\begin{array}{c} \theta_{k}=\tan^{-1}\left[\frac{\mathrm{angle}\left(g_{k}\right)}{\mathrm{angle}\left(f_{k}\right)}\right],\\ \phi_{k}=\sin^{-1}\left\{\frac{\lambda }{2\pi }\mathrm{sqrt}\left[\mathrm{angle}{\left(g_{k}\right)}^{2}+\mathrm{angle}{\left(f_{k}\right)}^{2}\right]\right\}, \end{array}$$

where angle(·) is the operator for getting phase angle. In conclusion, the steps of the GA-ESPRIT algorithm are:

1. The original data received from three subarrays are integrated into the measurement model of the whole array according to (25);
2. Calculate the covariance matrix $\hat{R}$, and then the ED in GA of $\hat{R}$ is performed and the signal subspace $U_{s}$ can be obtained by the larger eigenvalues;
3. According to (30), the signal subspace $U_{s}$ of the whole array is divided into three subspaces $U_{s1}$, $U_{s2}$ and $U_{s3}$;
4. $\Psi_{x}$ and $\Psi_{y}$ can be obtained using TLS in GA, and details can be found in (34)–(37);
5. The ED of $\Psi_{x}$ and $\Psi_{y}$ is performed to obtain matrices F and G;
6. The eigenvalues are matched in line with (38) and then taken them into Equation (39) to calculate *K* pairs direction angles.

**Figure 4:**
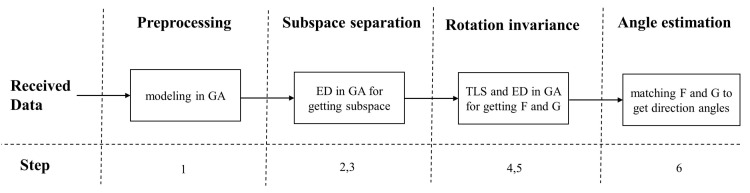
Logic flow diagram of GA-ESPRIT.

Further, the corresponding relationship between the logic flow and steps of GA-ESPRIT is shown in Figure 4.

### 3.4. Complexity Analysis

As discussed in [21,22,27], the estimation of the data covariance matrix is an important factor to illustrate the complexity of ESPRIT algorithm and another one is ED, because they imply many repetitive operations and results, which mean heavy computational burden and memory requirements. Thus, we evaluate the time complexity of the two processes and space complexity in terms of real value memory requirements.

Suppose that an array composed of *M* vector sensors, and *N* snapshots are taken. LV-ESPRIT [13] and GA-ESPRIT consider six-component measurements of each vector sensor, whereas Q-ESPRIT [25] only records two-component measurements (electric field on *x*-axis and *y*-axis.); therefore, we compare the complexity between LV-ESPRIT and GA-ESPRIT. The output of each vector sensor for each signal consists of six complex numbers in LV-ESPRIT, while GA-ESPRIT only has one multi-vector with vector and bivector parts.

The geometric product of two multi-vectors received by the array output implies 36 real multiplications [21], which is nine times as many real multiplications as two complex numbers. As mentioned in Section 2, the ED of a $G_{3}$ matrix is calculated by its CRM; therefore, the time complexity of the two algorithms is shown in Table 1. As for space complexity, the memory requirements of a real number is used to measure [21]. In the following two tables, CM is the Covariance Matrix, R represents real number.

The complexity comparison of these two algorithms can be found in Table 2, where CM and R represent covariance matrix and real number, respectively. Observing the time complexity in Table 2, it is not difficult to find that the computational burdens of CM and ED in GA-ESPRIT are a quarter and 1/27 of these in LV-ESPRIT, respectively. As for space complexity, GA-ESPRIT achieves such a significant reduction, more than 1.5 times compared to LV-ESPRIT, which means that the memory pressure is alleviated, especially for the large data size. The reason for the above comparison results is the natural advantage of GA matrix operations. In detail, because the six-dimensional measurement data in LV-MODEL (stored as 12 real numbers) are mapped into a multi-vector in the G-MODEL (stored as six real numbers), the amount of calculation will be reduced to varying degrees with different matrix operations, which will also bring fewer data storage requirements. The superior description and calculation ability of GA for multi-dimensional signals make GA-ESPRIT a very notable method for direction finding.

## 4. Simulation Results and Analysis

In this section, we simulate and analyze the proposed GA-ESPRIT based on DPULAs with d=λ/2, discuss its feasibility and performance compared with LV-ESPRIT [14] (in complex number field) and Q-ESPRIT [24] (in quaternion field). The estimation accuracy is evaluated by Root Mean Square Error (RMSE), which is calculated by the average of 200 Monte Carlo simulation experiments.

The RMSE of DOA estimation is defined as

(40) $$\mathrm{RMSE}=\frac{1}{K}\sum_{k=1}^{K}\sqrt{\frac{1}{200}\sum_{k=1}^{200}{\left[(\Delta \theta_{k}\right]}^{2}+{\left(\Delta \phi_{k}\right)}^{2}]},$$

where *K*, $\Delta \theta_{k}$ and $\Delta \phi_{k}$ denote the number of incident signals and errors between the result calculated by DOA algorithm and direction angle initially defined in the experiment, respectively.

In actual applications, the sensor model errors [27,41,42,43] cannot be ignored, which main include sensor-position error, gain error and phase error. The sensor-position error, as defined in [27], is the error between the actual position and the ideal position of each vector sensor. In the simulation experiment, the sensor-position error is modeled as additive noise with uniform distribution in a certain range, that is,

(41) $${\bar{k}}_{m}=k_{m}+d\sqrt{P_{pe}}{\left[\varepsilon_{mx},\varepsilon_{my},\varepsilon_{mz}\right]}^{T}$$

where ${\bar{k}}_{m}$ and $k_{m}$ are the actual position and ideal position of the mth sensor in vector-sensor array, respectively. $\varepsilon_{mx}$, $\varepsilon_{my}$ and $\varepsilon_{mz}$ are uniformly distributed noise terms. $P_{pe}$ represents the perturbation power of sensor-position error and the larger $P_{pe}$ means the greater deviation of the sensor from its ideal position. Further, referring to [43], the array output with the gain and phase error is denoted by

(42) B(t)=(I+ΠΞ)CS(t)+N(t),

where

$$\begin{array}{c} \Pi =\mathrm{diag}\left(\eta_{1},\eta_{2},\ldots ,\eta_{2M+2}\right),\\ \Xi =\mathrm{diag}\left(\exp\left(e_{123}\xi_{1}\right),\ldots ,\exp\left(e_{123}\xi_{2M+2}\right)\right), \end{array}$$

in which $\eta_{i}$ and $\xi_{i}$ (i=1,2,3,…,2M+2) are gain error and phase disturbance, respectively. In this paper, we also model them as additive noise. In addition, the six components of all EMVSs are added with noise according to the Signal-to-Noise ratio (SNR) in the following experiments. The SNR is defined as $SNR=10lg(P_{s}/P_{n})$, in which $P_{s}$ and $P_{n}$ are the power of signal and noise on each component, respectively.

**Figure 5:**
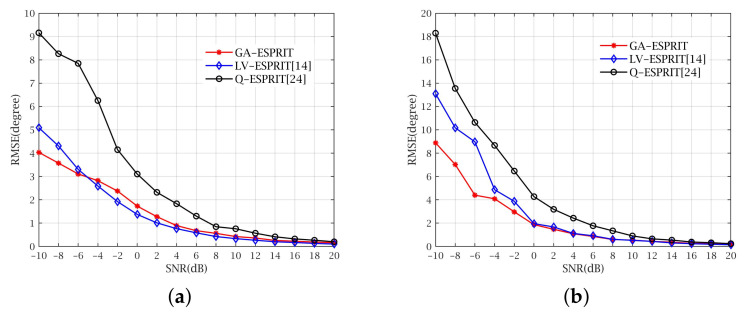
RMSE versus SNR with different noise. (**a**) RMSE versus SNR with uncorrelated noise. (**b**) RMSE versus SNR with correlated noise.

In the first experiment, we consider three far-field, narrow-band and uncorrelated signals with parameters Γ $=\left\{160^{\circ },80^{\circ },35^{\circ },-60^{\circ }\right\}$, $\left\{60^{\circ },50^{\circ },35^{\circ },60^{\circ }\right\}$ and $\{20^{\circ },110^{\circ }$, $45^{\circ },80^{\circ }\}$ with respect to Signal-to-Noise ratio (SNR) vary −10 dB to 20 dB in two different cases. In addition, we set M=7 and the snapshot number is 200. The aim of the first experiment was to examine the performance of GA-ESPRIT, LV-ESPRIT and Q-ESPRIT under different noise statistical characteristics. Figure 5a shows the estimation results of three algorithms when ideal Gaussian white noise is added, whereas, the noise in Figure 5b is related. It can be concluded that the three algorithms have very close accuracy of calculating DOA at high levels of SNR from Figure 5a,b, while with the lower SNR, GA-ESPRIT has higher accuracy over the other two and can achieve remove the correlation of noise partially.

**Figure 6:**
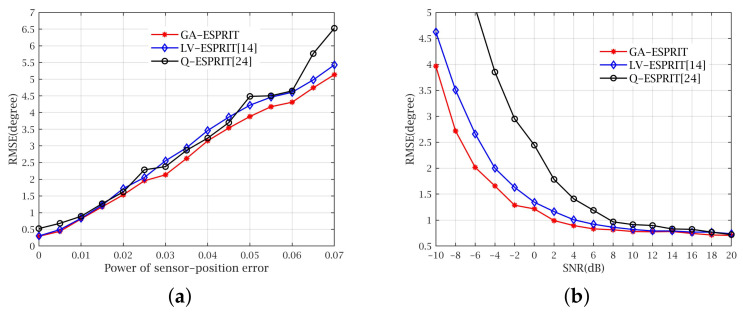
RMSE with sensor-position error. (**a**) RMSE versus the power of sensor-position error. (**b**) RMSE versus SNR in the presence of sensor-position error.

In the second experiment, we compare the performance of GA-ESPRIT, LV-ESPRIT and Q-ESPRIT when the sensor-position error exists. Assume that two signals with Γ $=\left\{58^{\circ },77^{\circ },35^{\circ },-60^{\circ }\right\}$ and $\left\{136^{\circ },50^{\circ },35^{\circ },60^{\circ }\right\}$ impinge on a DPULA with M=9. Figure 6a shows the performance of the three algorithms when sensor-position error exists with different intensities. Meanwhile, we set SNR to 10 dB and the snapshot number is 200. The sensor-position error of the array sensor is changed by the value of $P_{pe}$, whose range is 0–0.07. It can be seen in Figure 6b that we fix $P_{pe}=0.02$ to observe the estimation of the three algorithms by altering SNR from −10 dB to 20 dB. Figure 6a,b both imply that accuracy of GA-ESPRIT is highest in the presence of the sensor-position error, so the conclusion is that GA-ESPRIT has the strongest robustness against sensor-position errors among the three algorithms.

**Figure 7:**
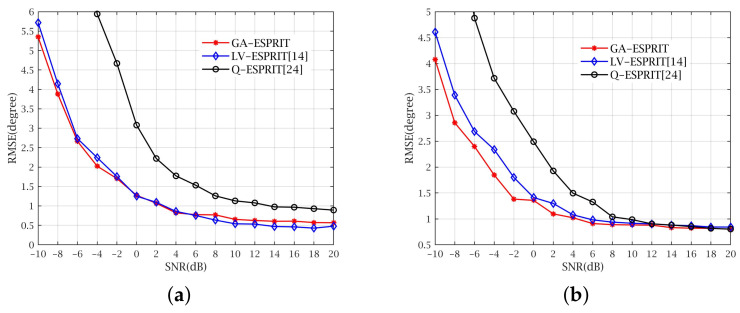
RMSE with gain or phase error. (**a**) RMSE versus SNR in the presence of gain error. (**b**) RMSE versus SNR in the presence of phase error.

The third experiment is also designed for two cases. Case one is that only gain error exists (see Figure 7a), while for case two, only phase error exists (see Figure 7b). Other conditions are the same as experiment two except that there is no position error. The gain error is constructed by the random numbers, whose mean value is 1 and variance is 0.2, and the phase error is constructed by the random numbers with zero-mean and 0.005 variance. We can learn from Figure 7a,b that, whether there is gain error or phase error, GA-ESPRIT can maintain the estimation accuracy very well, especially in low SNR.

In general, it is because Q-ESPRIT only takes part of the array output information into consideration that makes large RMSE. The reason for LV-ESPRIT’s poor accuracy in the face of the sensor-model error would be that its “long vector” destroys the orthogonality of the signal components. The improvement of detection robustness of GA-ESPRIT largely results from the fact that it can effectively preserve the orthogonality of the signal components and guarantee the completeness of the information.

## 5. Conclusions

In this paper, considering that the GA representation contains physical interpretations and complete information of incident signals, we use the idea of the traditional ESPRIT algorithm to find multiple EM signals in the direction finding method in GA. In particular, the model for DPULAs was built in GA and GA-ESPRIT was successfully derived using new calculation rules to achieve the two-dimensional DOA estimation. Compared with the previous ESPRIT algorithms, due to the robustness to sensor-model error and correlated noise, our proposed approach has potential in many practical situations, such as military radar in difficult environments. According to the experimental results, we have confirmed that the GA-ESPRIT has improved accuracy in two-dimension DOA estimation and can resist environmental interference to some extent. More importantly, the proposed algorithm achieves a reduction of more than 1/3 of the memory requirements while the time complexity is also greatly decreased.

Future works on the GA-ESPRIT will include polarization parameter estimation by optimizing matrix operations in GA and the ability of DOA recognition when facing coherent EM signals. It is expected that the proposed GA-ESPRIT will be an efficient DOA estimator.

## Figures and Tables

**Table 1:** The multiplication rule in $G_{3}$.

	1	$e_{1}$	$e_{2}$	$e_{3}$	$e_{12}$	$e_{23}$	$e_{13}$	$e_{123}$
1	1	$e_{1}$	$e_{2}$	$e_{3}$	$e_{12}$	$e_{23}$	$e_{13}$	$e_{123}$
$e_{1}$	$e_{1}$	1	$e_{12}$	$e_{13}$	$e_{2}$	$e_{123}$	$e_{3}$	$e_{23}$
$e_{2}$	$e_{2}$	$-e_{12}$	1	$e_{23}$	−$e_{1}$	$e_{3}$	−$e_{123}$	−$e_{13}$
$e_{3}$	$e_{3}$	−$e_{13}$	−$e_{23}$	1	$e_{123}$	−$e_{2}$	−$e_{1}$	$e_{12}$
$e_{12}$	$e_{12}$	−$e_{2}$	$e_{1}$	$e_{123}$	−1	$e_{23}$	−$e_{23}$	−$e_{3}$
$e_{23}$	$e_{23}$	−$e_{123}$	−$e_{3}$	$e_{2}$	−$e_{13}$	−1	$e_{12}$	−$e_{1}$
$e_{13}$	$e_{13}$	−$e_{3}$	−$e_{123}$	$e_{1}$	$e_{23}$	−$e_{12}$	−1	$e_{2}$
$e_{123}$	$e_{123}$	$e_{23}$	−$e_{13}$	$e_{12}$	−$e_{3}$	−$e_{1}$	$e_{2}$	−1

**Table 2:** Complexity of GA-ESPRIT and LV-ESPRIT.

Method	Time Complexity		Space Complexity		
	CM	ED	CM (R)	Eigenvalue (R)	Eigenvectors (R)
LV-ESPRIT	$O\left\{N\cdot {\left(6M\right)}^{2}\right\}$	$O\left\{{\left(6M\right)}^{3}\right\}$	72$M^{2}$	6*M*	36$M^{2}$
GA-ESPRIT	$O\left\{9N\cdot M^{2}\right\}$	$O\left\{{\left(2M\right)}^{3}\right\}$	8$M^{2}$	2*M*	64$M^{2}$

## Data Availability

Not applicable.
